# Surgical and Functional Outcomes of Posterior Cordotomy and Partial Arytenoidectomy with CO_2_ LASER in the Treatment of Bilateral Vocal Cord Immobility: A Single Institution Experience

**DOI:** 10.3390/jcm13133670

**Published:** 2024-06-24

**Authors:** Virginia Fancello, Andrea Migliorelli, Isotta Campomagnani, Federica Morolli, Francesco Stomeo, Andrea Ricci-Maccarini, Massimo Magnani, Marco Stacchini

**Affiliations:** 1 ENT & Audiology Unit, Department of Neurosciences, University Hospital of Ferrara, 44124 Ferrara, Italy; 2 ENT Department, M. Bufalini Hospital, 47521 Cesena, Italy

**Keywords:** bilateral vocal cord immobility, posterior cordotomy, partial arytenoidectomy, vocal cord paralysis

## Abstract

**Background/Objectives**: The purpose of this study is to investigate surgical and functional outcomes of patients affected by bilateral vocal cord immobility (BVCI) and treated with posterior cordotomy and partial arytenoidectomy. **Methods**: We performed a retrospective analysis on pre- and postoperative findings on a series of 27 patients affected by BVCI and treated with posterior cordotomy and partial arytenoidectomy from January 2017 to January 2022. Perceptual voice evaluations were performed using the GRBAS scale. The patients were requested to estimate the level of voice handicap experienced in their life using the Italian version of Voice Handicap Index 10 (VHI 10) questionnaire, while swallowing difficulties were self-evaluated through the Italian version of the Eating Assessment Tool (EAT-10) questionnaire. **Results**: Respiratory distress was evaluated according to the American Medical Research Council Dyspnoea Scale (MRC_DS) before and 1 year after the surgery. The mean of the preoperative values was 3.86 (±0.4), while 1 year after the procedure, we witnessed a significant (*p* ≤ 0.001) improvement, with a mean value of 1.09 (±0.9). After surgery, an overall worsening in voice quality was perceived, with a worsening in the GRBAS score. In contrast, the VHI10 does not show a statistically significant worsening. EAT 10 did not demonstrated worse scores after the surgery; rather, it showed a trend of improvement (preoperative EAT10 5.5 ± 5.8, postoperative 3.3 ± 2.9, *p* = 0.064). **Conclusions**: According to our results, posterior cordotomy plus partial arytenoidectomy is an effective procedure that provides stable and rapid respiratory improvement whilst preserving swallowing and the self-perception of voice quality.

## 1. Introduction

Bilateral vocal cord immobility (BVCI) is a rare condition that can result in stenosis of the glottis with potentially fatal airway compromise. A narrowing at the level of the vocal cords increases airway resistance, resulting in a disparity between respiratory efforts and the effective amount of air delivered to the lungs. This scenario, especially during physical activities, is the source of the dyspnoea experienced by patients. 

Neurogenic BCVI is caused by a central lesion or vagal or direct recurrent laryngeal nerve (RLN) damage. 

A large share of BCVI results from complications of thyroid surgery, which carries a risk of permanent bilateral vocal cord palsy ranging from 1.55‰ to 8.7‰, with lower risk in studies with neuromonitoring [1].

According to these data, bilateral permanent nerve injury, usually a result of total transection of the nerve or the disruption of its vascular supply, is considered a rare event. 

Iatrogenic lesions of RLN appear to show preferential damage for the nerve branches directed to the posterior cricoarytenoid muscle; thus, when the fibres for the only abductor muscle of the larynx are injured, the vocal cords fail to open during inspiration [2,3].

Mechanical blockage of the cricoarytenoid joint, related to arytenoids ankylosis, interarytenoid scarring, or interarytenoid web, can also cause BVCI. These peculiar conditions result from traumatic or prolonged endotracheal intubation, laryngeal trauma, radiation therapy, and/or autoimmune conditions such as rheumatoid arthritis and caustic damage.

During the SARS-CoV-2 outbreak, Italy became one of the epicentres of the pandemic. The rapid escalation of new cases left clinicians unprepared, and especially in the early stages, consensus guidelines on tracheostomy in COVID-19 patients were absent. At first, tracheostomies were often postponed because of the contagion risks and the unclear advantages [4,5], with a consequently expected high rate of prolonged intubation and related complications such as vocal cord immobility.

The clinical presentation of BVCI is characterized by dyspnoea, stridor, dysphonia, and aspiration of various degrees of severity that may occur immediately after the injury or develop progressively over time.

In the event of acute dyspnoea, an emergency tracheostomy can be performed to relieve respiratory distress. Moreover, it is the first approach in cases of BCVI of iatrogenic origin since temporary impairment is reported in around 79–87.5% of cases, and signs of recovery may occur even after 12 months post-injury [2,6].

Though tracheostomy is essential in BVCI management, it carries a high incidence of serious complications, and it is rarely accepted as a permanent option due to the significant impact on life.

Many glottic widening procedures have been developed throughout the years to improve the breathing efficiency of BVCI patients by creating a static enlarged airway.

In 1910, JW Gleitsmann reported the cordectomy as an alternative treatment to permanent tracheostomy to enlarge the glottis in case of bilateral vocal paralysis [7].

In his research, he first described the surgical dilemma between voice and breathing, observing that a large share of patients with BCVI would be willing to lose their voice to relieve the respiratory distress and close the tracheostomy. 

Since then, glottic widening procedures have evolved and changed, with the purpose of respecting the laryngeal structure whilst preserving voice quality and sphincteric laryngeal function as much as possible. 

With the advent of CO_2_ LASER and its application in laryngeal surgery, less invasive endoscopic approaches were proposed by Ossoff, Dennis and Kashima, and Crumley [8,9,10].

Later, Remacle et al. described the combination of subtotal arytenoidectomy with the preservation of a thin posterior portion of the cartilage associated with posterior cordotomy extended to the false cord. They also demonstrated how the use of the continuous super pulse LASER modality decreased surgery time [11,12].

Additional surgical techniques for posterior cordotomy using radiofrequency microelectrodes and endoscopy have also been described recently [13,14].

However, static glottic widening procedures with CO_2_ LASER, such as posterior cordotomy and partial arytenoidectomy, represent the gold standard of treatment.

Nowadays, these procedures are performed worldwide, although they modify the morphology of the laryngeal framework, with consequences on swallowing, voice quality, and physical performance.

In addition, despite surgical advances, a variable percentage of patients require revision surgery to remove granulation, fibrin, and scar tissue and to enlarge a narrowed airway. 

The aim of this study is to investigate the surgical and functional outcomes of patients affected by BCVI and treated with posterior cordotomy and partial arytenoidectomy at the Voice Center of Bufalini Hospital, Cesena, Italy. 

## 2. Materials and Methods

We performed a retrospective analysis on pre- and postoperative findings on a series of 27 patients affected by BVCI and treated with posterior cordotomy and partial arytenoidectomy in our hospital over a period of time of 5 years, from January 2017 to January 2022. Demographic data, including age, sex, smoking habit, and BMI, were collected. The objective outcome measures included flexible digital videolaryngoscopy, performed with Video Rhinolaryngoscope Olympus ENR-VQ (Olympus Medical System Corporation, Tokyo, Japan), maximum phonation time (MPT), decannulation time (length of time between first glottic widening procedure and decannulation). MPT, an aerodynamic parameter of voice production, was used to indirectly evaluate glottal competence. It consists of the prolonged vocal emission of an /a:/ for as long as possible after maximum inspiration. Three attempts are required, the longest being selected for comparison to the norm. It is simple to perform but can be easily influenced by fatigue and lung capability. Subjective assessments of voice quality were performed for both medical staff and patients. Perceptual voice evaluations were performed by an experienced phoniatrician using the GRBAS scale (G, Overall Grade; R, Roughness; B, Breathiness; A, Asthenia; and S, Strain) pre- and postoperatively. Each parameter was rated according to a 4-point scale (0 = normal; 1 = slight disturbance; 2 = moderate disturbance; 3 = severe disturbance) [15].

The patients were requested to estimate the level of voice handicap experienced in their life using the Italian version of Voice Handicap Index 10 (VHI 10) questionnaire, while swallowing difficulties were self-evaluated through the Italian version of the Eating Assessment Tool (EAT-10) questionnaire [16,17].

The degree of subjective respiratory distress was assessed using the Medical Research Council (MRC) dyspnoea scale, a five-item self-assessment tool originally developed to identify patients affected by chronic obstructive pulmonary disease. The MRC-DS has been demonstrated to be sensitive to the presence of glottic stenosis, being especially responsive to changes after treatment [18,19].

The surgical technique adopted was a combination of the posterior cordotomy and the subtotal arytenoidectomy, as described by Remacle et al., with CO_2_ LASER (Lumenis Acupulse^®^, Yokneam, Israel) using 9–10 watts of power in the continuous super pulse mode. 

**Figure 1:**
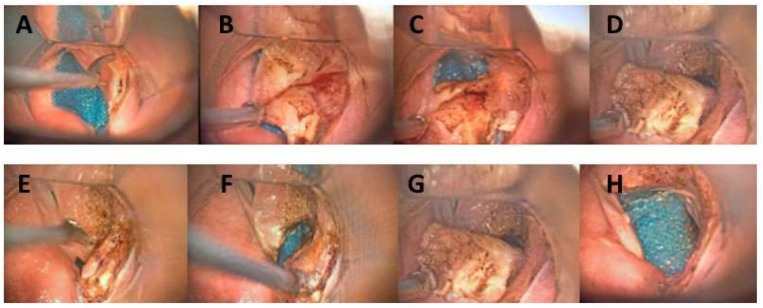
Intraoperative images. (**A**–**D**) Unilateral partial vestibulotomy to remove the posterior third of the right false vocal fold. (**E**) Incision of the mucosa of the vocal cord to expose the arytenoid vocal process. (**F**,**G**) Incision of the arytenoid vocal process and lateral extension of the vocal cord incision. (**H**) Monobloc excision of the posterior cordotomy and partial arytenoidectomy.

Exuberant scarring and/or oedema, false cord hypertrophy, and arytenoid prolapse are well-known limitations of isolated classic posterior cordotomy. To avoid these inconveniences, a partial posterior vestibulotomy is always performed alongside a partial arytenoidectomy, which is required to avoid posterior airway narrowing and subsequent inspiratory dyspnoea (Figure 1). Other key points of the procedure are the preservation of the arytenoid posterior portion, to provide airway protection, and the preservation of the anterior and medial thirds of the vocal cord original body layer, to limit the negative effects on phonation, thus maintaining discrete voice quality. Prophylactic antibiotic therapy was routinely administrated. The aim of the surgery was the creation of an effective airway and subsequent decannulation. 

A descriptive analysis of all variables with their frequencies was performed. The data collected are reported as mean ± standard deviation (SD) and as percentage to express continuous variables and categorical variables, respectively. Statistical significance was reported at the alpha level of 0.05 (α = 0.05). A *p*-value below 0.05 was considered significant (*p* < 0.05). A two-tailed unpaired Student’s *t*-test and the Wilcoxon signed-rank test were used to compare preoperative and postoperative results. A linear regression test was used to analyse the factors that could influence the decannulation time. Data analysis was performed using Microsoft Excel (v16.47.1, Microsoft, Redmond, WA, USA) and the Statistical Package for Social Sciences (SPSS v28.0.1.1, IBM, Armonk, NY, USA). 

## 3. Results

From January 2017 to January 2022, 27 patients underwent posterior cordotomy plus partial arytenoidectomy with CO_2_ LASER. 

Overall, 74% of patients were female, with the mean age at the onset of BVCI being 54.6 years old (range 23–80 years). Demographic data are summarized in Table 1. Vocal cord impairment was categorized according to the Nouraei and Sandu classification of BCVI, which identifies various types of obstruction [20]. In our series of patients, 74% were classified as 1a (peripheral denervation), 11% as 1b (central pathology, specifically acute cerebral stroke), and 15% as 2b (cricoarytenoid joint ankylosis following prolonged intubation, which occurred in two cases during COVID 19 infection). As expected, most of subjects (15/27) had BVCI following thyroid surgery. 

### 3.1. Dyspnoea 

**Chart 1:**
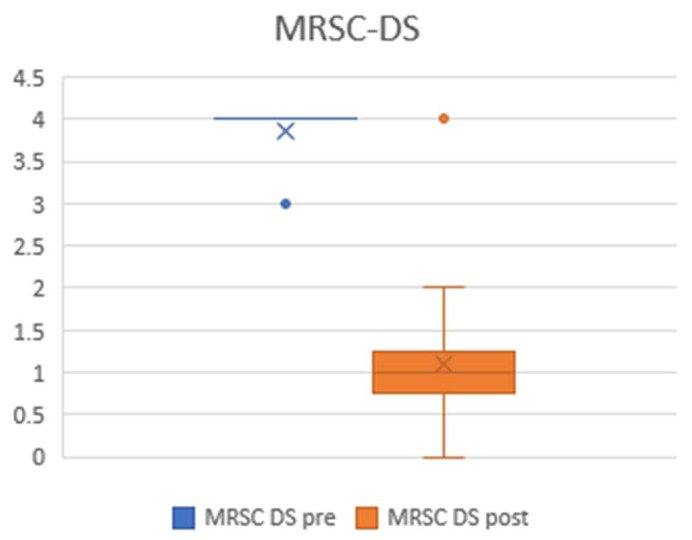
Plots showing pre- and postoperative MRSC-DS values. The preoperative mean was 3.86 (±0.4), and the postoperative mean was 1.09 (±0.9).

Respiratory distress was evaluated according to the American Medical Research Council Dyspnoea Scale (MRC_DS) before and 1 year after the surgery. The mean for the preoperative values was 3.86 (±0.4), while 1 year after the procedure, we witnessed a significant (*p* ≤ 0.001) improvement, with a mean value of 1.09 (±0.9) (Chart 1).

### 3.2. Decannulation

Eleven patients were tracheostomy-dependent at presentation. Since a large share of the patients were referred from other regions of Italy, a vigilant approach with elective tracheostomy, performed simultaneously to the treatment, was chosen to avoid respiratory complications after discharge while awaiting the stabilization of the outcomes and to facilitate postoperative airway management. Before decannulation, an examination of the glottis was performed with flexible fibreoptic nasopharyngolaryngoscopy through a traditional trans-nasal approach, as well as through a tracheostomic route. 

The average time from glottic surgical intervention to decannulation was 2.5 months ± 2.3. 

Minimal office-based revision surgery was offered to six subjects who developed granuloma (4/6) and fibrine excess (2/6), carried out by means of fibreendoscopic phonosurgery (FEPS) under local anaesthesia, to speed up the healing process, favour the absorption/removal of fibrin and granulation tissue, and spare the patients from additional distress.

Revision surgery under general anaesthesia was required to enlarge the glottic airway with CO_2_ LASER in five subjects with re-stenosis of the airway; only one patient required surgery on the contralateral vocal fold. 

After the revision, an adequate glottic airway was maintained long term, and the patients experienced successful decannulation. 

Among the diverse variables considered, including BMI, smoking, aetiology of BCVI, the timing of decannulation was influenced only by the age of the subjects at the onset of the palsy (*p* = 0.026) and the age at the time of treatment (*p* ≤ 0.001). 

**Figure 2:**
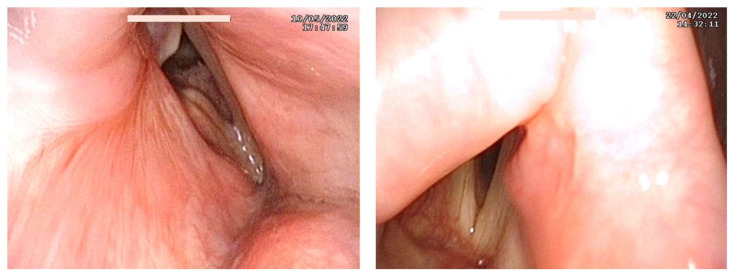
Frames from postoperative laryngoscopy.

Endoscopic evaluation after 1 year from the procedure demonstrated an improved and stable glottic inlet in all patients (Figure 2).

However, to date, two subjects who experienced BCVI after a stroke have not experienced decannulation, in consideration of their general condition; despite this, they are able to keep a non-cuffed cannula closed, even during activities. 

Therefore, their data were not included in further analysis. 

### 3.3. Voice

The outcome measures employed to evaluate the voice were VHI10, GRBAS, and MPT, used before the surgery and 1 year after. 

**Chart 2:**
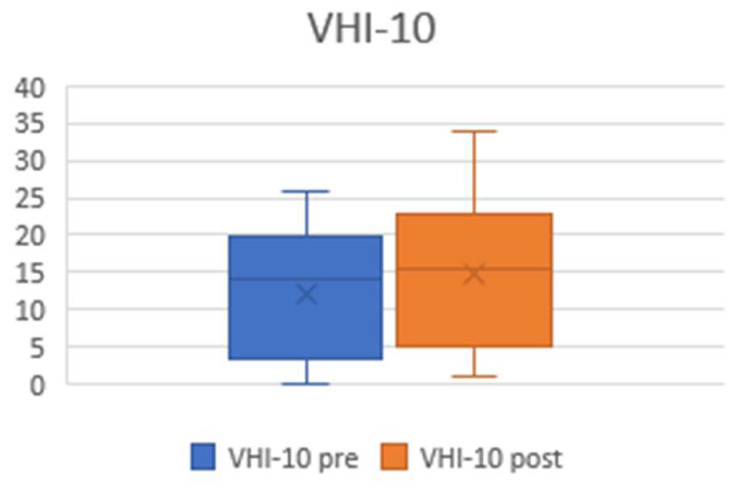
Plots showing pre- and postoperative VHI10 values. The preoperative mean was 12.1 (±8.5), and the postoperative mean was 14.8 (±9.8).

The self-assessment questionnaire, VHI 10, was administrated prior to surgery and after at least 12 months. The patients appreciate the treatment and noted that it had a positive impact on their quality of life, despite the deterioration of their voice. Thus, the VHI 10 after treatment did not deteriorate as expected (Chart 2).

The mean preoperative VHI 10 score was 12.1 (±8.5); the mean postoperative VHI 10 score was 14.8 (±9.8). Overall, the VHI 10 increased minimally (*p* = 0.137), without statistical significance. 

Despite voice deterioration, all decannulated patients stated that they would have undergone the same procedure again if they had known the outcome in advance.

Pre- and postoperative GRBAS was scored by an experienced phoniatrician. After surgery, an overall worsening in voice quality was perceived, along with a worsening in GRBAS score. Breathiness was the main affected parameter, with a statistically significant increased score (Table 2). 

**Chart 3:**
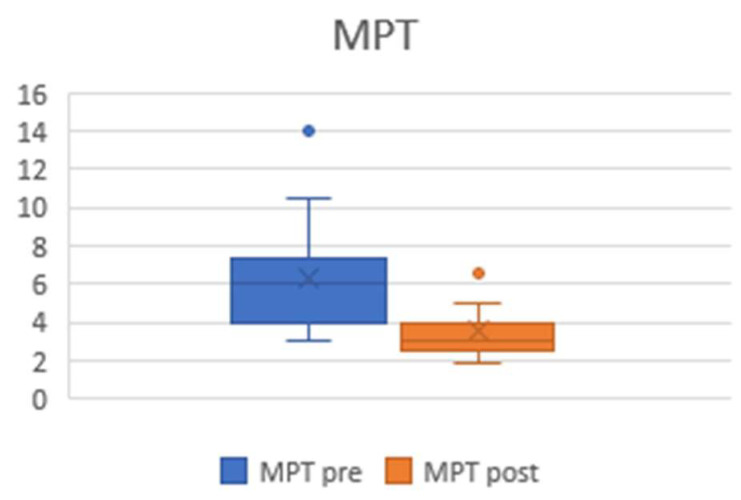
Plots showing pre- and postoperative MPT values. The preoperative mean was 6.4 s (±2.7), and the postoperative mean was 3.7 s (±1.6).

Maximum phonation time (MPT) reflects glottic competence. As expected, MPT deceased significantly (*p* ≤ 0.001) after the glottic widening procedure, with a mean preoperative value of 6.4 s (±2.7) and a mean postoperative value of 3.7 s (±1.6) (Chart 3).

### 3.4. Swallowing

**Chart 4:**
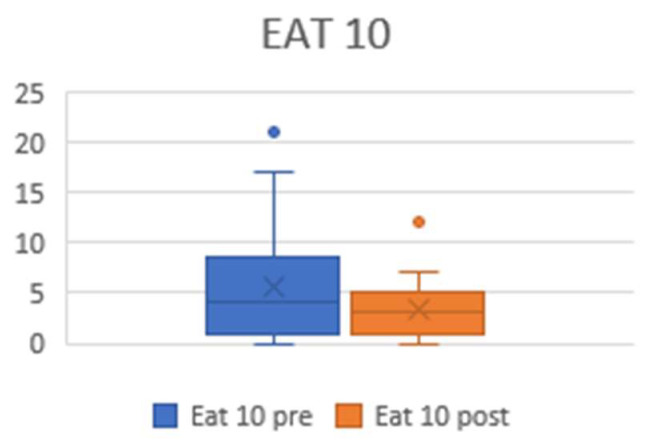
Plots showing pre- and postoperative EAT10 values. The preoperative mean was 5.5 (±5.8), and the postoperative mean was 3.3 (±2.9).

The EAT 10 questionnaire was used to assess dysphagia before and 1 year after surgery. All patients underwent a course of swallowing exercises with speech and language therapists. At the end of the study, they all tolerated a regular diet of varying components and fluids. There were no documented cases of aspiration pneumonia. EAT 10 did not demonstrate worse scores after the surgery (Chart 4); rather, it showed a trend of improvement (preoperative EAT10 5.5 ± 5.8, postoperative 3.3 ± 2.9, *p* = 0.064) that may have been caused by the decannulation.

## 4. Discussion

The most frequent cause of BVCI in the current study was thyroid surgery (15 out of 27 cases), and the majority of patients (20 out of 27 subjects) were female. According to the literature, women are more likely than men to have thyroid disease, which increases the chance that they may need thyroid surgery and the subsequent likelihood of complications [21].

Among those, BVCI has a dramatic impact on life quality, as the larynx is a vital organ that governs breathing, swallowing, airway protection, and, in humans, voice production. All of these actions are possible thanks to the vocal cords’ ability to adapt their position dynamically to different activities, ensuring that the airway seals and opens at the appropriate time during phonation, breathing, lifting, and applying abdominal pressure.

Interestingly, two subjects reported mechanical BCVI following admission to the intensive care unit and prolonged endotracheal intubation during COVID 19 infection. Both patients had been already tracheostomized when this was brought to our attention. 

The posterior glottis is one of the most injured areas of the larynx during intubation, and the trauma may be the source of cricoarytenoid fixation, as was seen in these cases. During the SARS-COV-2 pandemic, there was a high rate of endotracheal intubation in patients who developed interstitial pneumonia [22,23].

Consequent complications, such as glottic stenosis, can be treated endoscopically with CO_2_ LASER, as reported in our experience, with successful outcomes.

Glottic widening procedures, including posterior cordotomy and partial arytenoidectomy, are designed to create a new static airway that does not vary throughout different laryngeal tasks. Thus, long-term complications are mainly related to impairment of the phonatory and sphincteric functions of the larynx. 

To provide an adequate laryngeal passage, deterioration of the voice is frequently the price to pay, because of the disruption to the vocal cords. Dysphonia may be a difficult symptom to accept since the voice is one of main tools for human social interaction. 

However, the findings of our study suggested that, despite the postoperative worsening in the GRBAS values, scored by experienced phoniatricians/laryngologists, posterior cordotomy plus partial arytenoidectomy provides good and stable respiratory outcomes without having a significant impact on patients’ perception of their voices, as demonstrated by a minimal (two-point) increase in mean VHI10 after surgery. 

The patients reported a clear relief of dyspnoea following the treatment, as observed by the dramatic reduction in MRC-DS, with possible positive effects on voice fatigue. This is probably the reason for the overall unchanged voice perception identified by self-assessment questionnaires, as was observed in our research, as well as in other published studies [24,25].

A general significant decrease in MPT (*p* ≤ 0.001) was detected after treatment. This result could be considered a sign of the procedure’s effectiveness, as MPT is a functional measure of glottic competence. The newly enlarged airway causes the dissipation of air through the glottis during phonation, with a consequently shorter MPT than those with a lower airflow volume. Accordingly, MPT is expected to decrease following posterior cordotomy +/− arytenoidectomy [26].

After surgery, the subjects may experience dysphagia. In our cohort, the EAT10 revealed a 2.5-point improvement in the mean postoperative scores. These data, despite not being statistically significant, may be related to the presence of tracheostomy in 40.7% of our patients before the treatment. The tracheostomy itself is a cause of altered subglottic pressure and impaired laryngeal sensitivity and mobility, ultimately resulting in some degree of dysphagia. Thus, decannulation may be key in swallowing amelioration. A few studies have suggested that, even when postoperative dysphagia is reported, the symptoms resolve without evidence of aspiration pneumonia, and long-term postoperative swallowing is comparable to preoperative swallowing [27,28,29,30].

Thus, immediately after treatment, it is critical to perform swallowing exercises which help to prevent adverse events such as aspiration and improve dysphagia, if present. 

Careful and thorough counselling is essential when dealing with BVCI patients in order to avoid disappointment or significant complaints about the outcomes. Before suggesting this type of surgery, the surgeon should consider the patient’s preferences and needs, taking into account age, comorbidities, and quality of life prior to BCVI.

Since this treatment results in permanent disruption of the laryngeal framework, it is important to always be aware of the difference between a temporary and permanent impairment of vocal cord mobility. Always wait a decent amount of time (from 6 months to 1 year) when permanent damage is not certain to allow for natural recovery, if that is possible [31].

Patients who are willing to undergo this surgery should be informed about the possibility of decannulation failure and of revision procedures.

The deterioration of one’s voice and swallowing ability has always been the major challenge for surgeons when performing this technique. However, according to our study, swallowing is a parameter that is not affected.

On the other hand, voice quality was perceived to deteriorate by the health professionals but not significantly so, according to the patients. This discrepancy, which has also been noted by other authors, needs to be explained to the patients because surgeons’ expectations may not match patients’ aspirations, regarding which the relief of respiratory distress represents the major concern.

Hopefully, in the near future, laryngeal reinnervation techniques will become the gold standard treatment for neurogenic BCVI, with the goal of re-establishing dynamic laryngeal functions and restoring physiological vocal cord movements, along with respiration and phonation without disruption of the anatomy [32].

However, patients not fit for prolonged general anaesthesia, those affected by mechanical BCVI, or those not willing to wait up to more than 1 year to appreciate the results of nerve regeneration will not be candidates for reinnervation, and traditional glottic widening procedures will still be their treatment of choice. 

## 5. Conclusions

According to our results, posterior cordotomy plus partial arytenoidectomy is an effective procedure that provides stable and rapid respiratory improvement whilst preserving swallowing and self-perception of voice quality.

## Figures and Tables

**Table 1:** Demographic data and patient characteristics.

Demographic Data	
Sex	Male 7 (26%) Female 20 (74%)
Age at the onset of BVCI	54.6 (23–80) (years old)
Etiology	1a (iatrogenic): 20 (74%) 1b (neurogenic): 3 (11%) 2 (postintubation): 4 (15%)
Smokers	Never 12 (44.5%) Currently smokers 5 (18.5%) Former smokers 10 (37%)
Body Mass Index (BMI)	26.6 ± 5.5 (18–39)
Timing BCVI onset–surgical treatment	5.9 years ± 8.7 (range 0.5–32)
Age at the time of surgery	60.4 ± 13.5 (range 27–81)
Side	Right 14 (53%) Left 13 (48%)
Tracheostomy	Previous 11 (41%) Consensual 16 (59%)
Decannulation	25 patients (93%) Timing 2.5 months ± 2.3 (range 1–9)

**Table 2:** Pre- and postoperative GRBAS scores.

	Preoperative	Postoperative	*p* Value
**G**	0.71 ± 0.6	1.38 ± 0.6	***p* = 0.013**
**R**	0.25 ± 0.5	0.46 ± 0.6	*p* = 0.305
**B**	0.29 ± 0.5	1.17 ± 0.6	***p* = 0.004**
**A**	0.46 ± 0.5	0.79 ± 0.6	*p* = 0.248
**S**	0.46 ± 0.7	0.46 ± 0.6	*p* = 0.782

## Data Availability

Data are contained within the article.
